# Targeting the Gut–Kidney–Heart Axis in Chronic Kidney Disease: The Mediterranean Diet as a Strategy to Reduce Uremic Toxins and Cardiovascular Risk

**DOI:** 10.3390/nu18091451

**Published:** 2026-04-30

**Authors:** Josipa Radić, Tina Bečić, Marijana Vučković, Ivana Jukić, Jonatan Vuković, Damir Fabijanić, Mislav Radić

**Affiliations:** 1 Department of Internal Medicine, Division of Nephrology, Dialysis and Arterial Hypertension, University Hospital of Split, 21000 Split, Croatia; josiparadic1973@gmail.com (J.R.); maryanchi.1@gmail.com (M.V.); 2 Department of Internal Medicine, School of Medicine, University of Split, 21000 Split, Croatia; jonatan.vukovic@mefst.hr; 3 Department of Cardiovascular Diseases, University Hospital of Split, 21000 Split, Croatia; tina.becic@gmail.com (T.B.); damirfabijanic62@gmail.com (D.F.); 4 Department of Internal Medicine, Division of Gastroenterology, University Hospital of Split, 21000 Split, Croatia; ivjukic@gmail.com; 5 Faculty of Health Sciences, University of Split, 21000 Split, Croatia; 6 Department of Clinical Propedeutics, School of Medicine, University of Split, 21000 Split, Croatia; 7 Department of Internal Medicine, Division of Rheumatology, Allergology and Clinical Immunology, University Hospital of Split, 21000 Split, Croatia

**Keywords:** chronic kidney disease, gut–kidney–heart axis, uremic toxins, indoxyl sulfate, *p*-cresyl sulfate, TMAO, Mediterranean diet, cardiovascular risk, gut microbiota, short-chain fatty acids

## Abstract

Chronic kidney disease (CKD) is associated with a markedly increased risk of cardiovascular (CV) morbidity and mortality that cannot be fully explained by traditional risk factors. Emerging evidence highlights the central role of the gut–kidney–heart axis, whereby gut microbiota dysbiosis promotes the generation and systemic accumulation of uremic toxins, including indoxyl sulfate (IS), *p*-cresyl sulfate (PCS), and trimethylamine *N*-oxide (TMAO). These metabolites contribute to endothelial dysfunction, oxidative stress, inflammation, and vascular remodeling, thereby accelerating CV disease progression in CKD. Dietary patterns represent a key modifiable factor influencing gut microbiota composition and metabolic activity. The Mediterranean diet, characterized by high intake of plant-based foods, dietary fiber, and polyphenols, and low consumption of red and processed meats, has emerged as a promising microbiota-targeted strategy. It promotes saccharolytic fermentation, enhances short-chain fatty acid production, and reduces proteolytic pathways responsible for uremic toxin generation. Accumulating evidence from observational studies, meta-analyses, and dietary intervention trials suggests that adherence to Mediterranean and plant-based dietary patterns is associated with reduced uremic toxin burden, improved renal outcomes, and lower CV risk in CKD populations. However, direct interventional evidence linking Mediterranean diet adherence to changes in specific uremic toxin levels remains limited. This narrative review summarizes current evidence on diet–microbiota interactions in CKD and highlights the Mediterranean diet as a biologically plausible strategy for targeting the gut–kidney–heart axis. Future well-designed randomized controlled trials (RCTs) are needed to confirm causal relationships and support clinical implementation.

## 1. Introduction: The Gut–Kidney–Heart Axis in Chronic Kidney Disease

Chronic kidney disease (CKD) represents a major global health burden and is strongly associated with increased CV morbidity and mortality, which cannot be fully explained by traditional risk factors alone. Increasing evidence suggests that non-traditional mechanisms, particularly those involving gut microbiota-derived metabolites, play a pivotal role in the development of CV complications in CKD [1,2,3,4,5]. The gut–kidney axis has emerged as a central pathophysiological pathway linking intestinal dysbiosis to systemic toxicity in CKD. Alterations in gut microbiota composition—characterized by reduced diversity and a shift toward proteolytic fermentation—promote the generation of uremic toxins that accumulate due to impaired renal clearance [6,7,8,9,10,11]. Importantly, these alterations are strongly influenced by dietary patterns, which represent a key modifiable factor shaping gut microbiota composition and metabolic activity in CKD. In addition, CKD-associated uremia contributes to intestinal barrier dysfunction, facilitating the translocation of microbial metabolites and inflammatory mediators into the systemic circulation [8,11]. Among gut-derived metabolites, protein-bound uremic toxins such as indoxyl sulfate (IS) and *p*-cresyl sulfate (PCS), as well as trimethylamine *N*-oxide (TMAO), have been identified as key contributors to CV damage in CKD [1,12]. These compounds exert multiple deleterious effects, including endothelial dysfunction, oxidative stress, vascular calcification, and myocardial fibrosis, thereby accelerating atherosclerosis and increasing the risk of adverse CV outcomes [1,2,3,4]. Importantly, the interaction between the gut, kidneys, and CV system is bidirectional and constitutes a complex gut–kidney–heart axis. CKD not only promotes dysbiosis and toxin accumulation but is also exacerbated by microbiota-derived metabolites that further impair renal and CV function [6,8,12,13]. This reciprocal interplay highlights the gut microbiota as a promising therapeutic target. In this context, dietary interventions have gained increasing attention as modulators of gut microbiota composition and metabolic activity. The Mediterranean diet, characterized by a high intake of plant-based foods, dietary fiber, and bioactive compounds, has been proposed as a key strategy to shift microbial metabolism toward a more favorable profile, potentially reducing the production of harmful uremic toxins and mitigating CV risk in CKD [14,15]. However, direct evidence linking Mediterranean diet adherence to reductions in specific gut-derived uremic toxins remains limited, underscoring an important gap in current research.

It is important to distinguish indirect evidence from mechanistic or observational studies from direct evidence from interventions related to CKD.

Despite growing evidence supporting the role of gut microbiota-derived metabolites in cardiovascular complications of CKD, several important gaps remain. Most available data are derived from observational studies or indirect mechanistic evidence, while direct interventional studies evaluating the impact of specific dietary patterns—particularly the Mediterranean diet—on circulating levels of individual uremic toxins such as indoxyl sulfate, *p*-cresyl sulfate, and TMAO remain scarce. Furthermore, the integration of diet–microbiota interactions within the broader gut–kidney–heart axis has not been systematically addressed in the context of cardiovascular risk modulation in CKD. Therefore, this narrative review aims to provide an updated and integrative synthesis of current evidence, with a particular focus on the Mediterranean diet as a microbiota-targeted strategy to reduce uremic toxin burden and cardiovascular risk in CKD.

**Figure 1:**
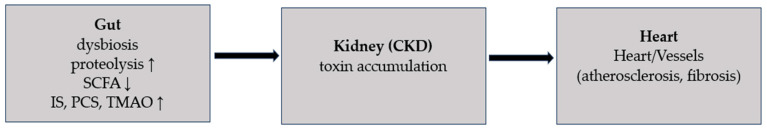
Schematic representation of the gut–kidney–heart axis in chronic kidney disease. Chronic kidney disease promotes gut microbiota dysbiosis and intestinal barrier dysfunction, leading to increased production of uremic toxins, including indoxyl sulfate (IS), *p*-cresyl sulfate (PCS), and trimethylamine *N*-oxide (TMAO). These metabolites contribute to endothelial dysfunction, inflammation, and vascular damage, ultimately contributing to increased cardiovascular (CV) risk. Abbreviations: CKD, chronic kidney disease; IS, indoxyl sulfate; PCS, *p*-cresyl sulfate; TMAO, trimethylamine *N*-oxide; SCFA, short-chain fatty acids.

The key mechanisms underlying the gut–kidney–heart axis are illustrated in Figure 1.

## 2. Literature Search Strategy

This narrative review is based on a comprehensive literature search conducted in major electronic databases, including PubMed, Scopus, Web of Science, and the Cochrane Library. Relevant studies published up to 2026 were identified using combinations of keywords such as “chronic kidney disease”, “gut microbiota”, “uremic toxins”, “indoxyl sulfate”, “*p*-cresyl sulfate”, “trimethylamine *N*-oxide”, and “Mediterranean diet”. The search was limited to articles published in English. Both original research articles and review papers were considered to provide a comprehensive overview of current evidence.

Inclusion criteria comprised studies investigating the relationship between dietary patterns, gut microbiota composition, uremic toxins, and cardiovascular outcomes in the context of chronic kidney disease. Exclusion criteria included studies not related to CKD, non-human studies without clear translational relevance, conference abstracts without available full text, and articles lacking sufficient methodological detail.

The study selection process involved initial screening of titles and abstracts, followed by full-text assessment of potentially relevant articles. Additional studies were identified through manual screening of reference lists of selected publications.

## 3. Gut Microbiota Dysbiosis in Chronic Kidney Disease

CKD is associated with profound alterations in gut microbiota composition and function, collectively referred to as dysbiosis. This condition is characterized by reduced microbial diversity and a shift from saccharolytic to proteolytic fermentation, driven by the accumulation of uremic toxins, metabolic acidosis, and dietary restrictions commonly observed in CKD patients [6,7,8,9,10]. As renal function declines, the retention of nitrogenous waste products alters the intestinal environment, favoring the expansion of proteolytic bacterial species that utilize amino acids as substrates, thereby increasing the production of toxic metabolites. These dysbiotic changes are further exacerbated by impaired intestinal barrier function and local inflammation, which contribute to increased permeability and systemic exposure to microbial-derived compounds [8,16]. Experimental and clinical studies have demonstrated that CKD is associated with significant shifts in microbial taxa, including a reduction in beneficial commensal bacteria and an overgrowth of urease-, uricase-, and indole-producing microorganisms [17,18]. A key consequence of this altered microbial metabolism is the enhanced generation of gut-derived uremic toxins, including IS, PCS, and other protein-bound solutes, which accumulate in the circulation due to reduced renal clearance [7,10,19]. These metabolites contribute not only to CKD progression but also to systemic inflammation and CV complications, highlighting the central role of gut microbiota dysbiosis in the pathophysiology of the gut–kidney–heart axis. These findings further support the role of dietary interventions, particularly fiber-rich and plant-based dietary patterns, in modulating gut microbiota composition and reducing toxin generation in CKD.

## 4. Gut-Derived Uremic Toxins and Cardiovascular Risk

Gut-derived uremic toxins represent a key mechanistic link between intestinal dysbiosis and the increased CV risk observed in patients with CKD. These metabolites originate from microbial metabolism of dietary substrates and accumulate in the systemic circulation due to impaired renal clearance, exerting multiple deleterious effects on vascular and cardiac function [1].

Although indoxyl sulfate (IS), *p*-cresyl sulfate (PCS), and trimethylamine *N*-oxide (TMAO) share common pro-atherogenic and pro-inflammatory effects, they act through partially overlapping but distinct molecular pathways. IS and PCS are primarily protein-bound uremic toxins derived from amino acid metabolism and exert potent effects on endothelial cells, vascular smooth muscle cells, and cardiomyocytes. In contrast, TMAO, derived from dietary nutrients such as choline and L-carnitine, is more strongly associated with alterations in lipid metabolism, platelet activation, and atherosclerotic plaque formation. While all three toxins contribute to oxidative stress, inflammation, and vascular dysfunction, their relative contributions and dominant mechanisms may differ depending on CKD stage, dietary exposure, and inter-individual variability in gut microbiota composition.

### 4.1. Indoxyl Sulfate and p-Cresyl Sulfate

IS and PCS are among the most extensively studied protein-bound uremic toxins derived from gut microbial metabolism of dietary amino acids, particularly tryptophan, tyrosine, and phenylalanine [20,21]. These compounds are generated through bacterial fermentation in the colon, followed by hepatic sulfation, and are poorly cleared by conventional dialysis due to their strong protein-binding properties. Both IS and PCS exert potent cytotoxic and pro-inflammatory effects on the CV system. Experimental studies have demonstrated that IS promotes oxidative stress, endothelial dysfunction, and vascular smooth muscle cell proliferation, contributing to vascular remodeling and stiffness, and is also implicated in the development of myocardial fibrosis in CKD [21,22,23]. Similarly, PCS has been shown to induce cardiomyocyte apoptosis, oxidative stress, and functional impairment, thereby contributing to adverse cardiac remodeling in CKD. Clinically, elevated circulating levels of IS and PCS are strongly associated with adverse CV outcomes, including increased risk of atherosclerosis, arterial stiffness, and mortality in CKD patients [24,25,26,27,28]. Moreover, recent studies have confirmed their predictive value for major adverse CV events and vascular dysfunction, reinforcing their role as key mediators of the gut–kidney–heart axis [29,30].

At the molecular level, gut-derived uremic toxins contribute to cardiovascular damage through several interconnected mechanisms. A central pathway involves activation of nuclear factor-κB (NF-κB), which promotes the transcription of pro-inflammatory cytokines and adhesion molecules, leading to endothelial activation and vascular inflammation. In addition, emerging evidence suggests that these toxins may activate the NLRP3 inflammasome, further amplifying inflammatory signaling and contributing to vascular injury.

Oxidative stress represents another key mechanism, characterized by an imbalance between reactive oxygen species (ROS) production and nitric oxide (NO) bioavailability. Uremic toxins have been shown to impair endothelial nitric oxide synthase (eNOS) activity, reducing NO production and promoting endothelial dysfunction. This ROS–NO imbalance contributes to vascular stiffness, impaired vasodilation, and progression of atherosclerosis.

Furthermore, mitochondrial dysfunction has been increasingly recognized as a critical component of toxin-mediated cellular injury. Uremic toxins can disrupt mitochondrial function, leading to impaired energy metabolism, increased ROS generation, and activation of apoptotic pathways in vascular and cardiac cells. These mechanisms collectively contribute to the progression of cardiovascular disease within the context of CKD.

### 4.2. Trimethylamine N-Oxide (TMAO)

TMAO is another major gut-derived metabolite implicated in CV risk, produced from dietary nutrients such as choline and L-carnitine through microbial metabolism and subsequent hepatic oxidation [12]. Diets rich in animal products, particularly red meat, provide substrates for TMAO production, highlighting the strong interaction between dietary patterns and gut microbiota in CKD. TMAO has been shown to promote atherosclerosis through multiple mechanisms, including enhanced foam cell formation, altered cholesterol metabolism, increased platelet reactivity, and vascular inflammation [2,31]. In CKD patients, circulating TMAO levels are significantly elevated due to reduced renal clearance, further amplifying its pathogenic effects. Importantly, numerous clinical studies and meta-analyses have demonstrated a strong association between elevated TMAO levels and increased risk of CV events and all-cause mortality in CKD populations [3,4,32]. These findings underscore the central role of TMAO as a diet–microbiota-derived mediator linking CKD to CV disease.

## 5. Mediterranean Diet: Composition and Effects on Gut Microbiota

The Mediterranean diet is characterized by a high intake of plant-based foods, including fruits, vegetables, legumes, whole grains, nuts, and extra virgin olive oil, combined with a low consumption of red and processed meats. This dietary pattern is rich in dietary fiber, polyphenols, and unsaturated fatty acids, which collectively exert beneficial effects on gut microbiota composition and metabolic activity [33,34,35,36]. Dietary fiber represents a key component of the Mediterranean diet and serves as a primary substrate for saccharolytic fermentation by gut microbiota. This process promotes the growth of beneficial bacterial taxa and enhances microbial diversity, counteracting the dysbiosis commonly observed in CKD [37,38]. In parallel, polyphenols derived from olive oil, fruits, and nuts exhibit prebiotic-like effects, selectively stimulating the proliferation of health-promoting microbial species while inhibiting the growth of pathogenic bacteria [39,40]. A key metabolic consequence of these microbiota changes is the increased production of short-chain fatty acids (SCFAs), including butyrate, propionate, and acetate. SCFAs play a crucial role in maintaining intestinal barrier integrity, modulating immune responses, and reducing systemic inflammation, thereby contributing to improved cardiometabolic health [37,39]. Importantly, the shift toward saccharolytic fermentation and SCFA production occurs at the expense of proteolytic pathways, which are responsible for the generation of harmful uremic toxins. Furthermore, the reduced intake of red meat and animal protein in the Mediterranean diet limits the availability of substrates for the production of gut-derived uremic toxins, such as IS, PCS, and TMAO. This dietary pattern therefore promotes a favorable microbial and metabolic profile, supporting its role as a microbiota-targeted strategy for mitigating toxin generation and CV risk in CKD [36,38].

Notably, the effects of the Mediterranean diet on gut microbiota and metabolic outcomes may vary significantly between individuals. This variability is influenced by baseline microbiota composition, CKD stage, dietary adherence, and host-related factors such as age, comorbidities, and metabolic status. As a result, responses to dietary interventions are often heterogeneous, highlighting the importance of personalized nutritional approaches in CKD management.

The strength of current evidence varies across different aspects of Mediterranean diet-mediated effects. There is strong evidence supporting its beneficial impact on CV outcomes and risk factor modification, primarily derived from large randomized controlled trials and meta-analyses. Evidence linking the Mediterranean diet to modulation of gut microbiota composition and increased SCFA production is emerging, supported by mechanistic studies and smaller clinical investigations. In contrast, direct interventional evidence demonstrating reductions in specific gut-derived uremic toxins, such as IS, PCS, and TMAO, remains limited.

Overall, the Mediterranean diet exerts its beneficial effects through coordinated modulation of gut microbiota composition and metabolic activity, promoting a shift toward saccharolytic fermentation, enhancing SCFA production, and reducing the generation of uremic toxins. These mechanisms collectively contribute to improved intestinal barrier function, reduced systemic inflammation, and attenuation of cardiovascular risk. Within the framework of the gut–kidney–heart axis, the Mediterranean diet represents a central and biologically plausible nutritional strategy for modulating disease progression and improving clinical outcomes in CKD.

## 6. Mediterranean Diet and Cardiovascular Protection: Evidence from Clinical Studies

The CV benefits of the Mediterranean diet are supported by a substantial body of clinical evidence, particularly from large-scale RCTs and prospective cohort studies. Among these, the PREDIMED (Prevención con Dieta Mediterránea) trial represents a landmark study demonstrating that adherence to a Mediterranean dietary pattern significantly reduces the incidence of major CV events, including myocardial infarction, stroke, and CV mortality [41]. In the PREDIMED trial, participants at high CV risk who followed a Mediterranean diet supplemented with extra virgin olive oil or nuts exhibited a significantly lower risk of major adverse CV events compared with those assigned to a low-fat control diet. These findings have been consistently supported by subsequent analyses and reviews, confirming the protective role of the Mediterranean diet in both primary and secondary CV prevention [42,43,44]. The cardioprotective effects of the Mediterranean diet are multifactorial and include improvements in lipid profiles, reduction in oxidative stress, modulation of inflammatory pathways, and enhancement of endothelial function. Importantly, emerging evidence suggests that these benefits are also mediated, at least in part, through modulation of gut microbiota composition and function [39]. This microbiota-mediated pathway may contribute to reduced production of pro-atherogenic metabolites and improved vascular homeostasis. Collectively, these findings reinforce the Mediterranean diet as a promising microbiota-targeted therapeutic strategy for reducing CV risk in patients with CKD.

## 7. Mediterranean Diet in CKD

The Mediterranean diet has gained increasing attention as a promising dietary strategy in patients with CKD, with potential benefits extending beyond CV protection to include modulation of renal function decline and overall disease progression. Growing evidence, including data from systematic reviews and meta-analyses, suggests that adherence to Mediterranean dietary patterns is associated with a slower decline in estimated glomerular filtration rate (eGFR), improved metabolic control, and reduced burden of cardiometabolic risk factors in CKD populations [33,34,35,36,45]. These effects are particularly relevant given the complex interplay between metabolic, inflammatory, and hemodynamic factors driving CKD progression. One of the key mechanisms underlying these benefits is the anti-inflammatory and antioxidative profile of the Mediterranean diet, which contributes to improved endothelial function and reduced intraglomerular hypertension. This, in turn, may attenuate structural and functional renal damage. In addition, dietary patterns rich in plant-based foods and low in saturated fats have been associated with improved blood pressure control and insulin sensitivity, both of which are critical determinants of CKD progression. Importantly, several clinical and observational studies have demonstrated that higher adherence to the Mediterranean diet is associated with lower levels of albuminuria, a well-established marker of glomerular injury and a strong predictor of both renal and CV outcomes [14,46]. Reduction in albuminuria may reflect improvements in endothelial integrity and decreased systemic inflammation, further supporting the renoprotective effects of this dietary pattern. As previously described, beyond traditional risk factors, the Mediterranean diet may provide additional benefits by modulating gut microbiota composition and metabolic activity.

These findings highlight the relevance of the Mediterranean diet within the framework of the gut–kidney–heart axis. Importantly, evidence from systematic reviews and meta-analyses in end-stage kidney disease populations further suggests that adherence to Mediterranean and other plant-based dietary patterns is associated with improved clinical outcomes [47] with emerging evidence extending these benefits to kidney transplant recipients [48], suggesting potential improvements in metabolic control, CV risk profile, and graft-related outcomes in this high-risk population. 

Recently published meta-analytic data on the Mediterranean diet in CKD show a modest improvement in eGFR and a reduction in C-reactive protein (CRP) as an anti-inflammatory effect, with clear component specificity, particularly in interventions that include high-phenolic extra virgin olive oil (CRP mean difference −0.79 mg/L, 95% CI −1.37 to −0.21; *p* = 0.008; *I*^2^ = 0%). The level of evidence remains low to moderate, as benefits regarding eGFR are observed in the subgroup with mild to moderate CKD (eGFR ≥ 45 mL/min/1.73 m^2^), while findings in advanced CKD are weak and inconsistent. Importantly, effect estimates were larger in observational studies than in RCTs, which could indicate potential confounding [49]. 

Adherence to the Mediterranean diet remains a critical determinant of its effectiveness in real-world settings. While the diet is generally well tolerated, maintaining long-term adherence may be challenging in CKD patients due to dietary restrictions, comorbidities, and cultural factors. Evidence suggests that higher adherence is consistently associated with more favorable renal and CV outcomes, whereas poor compliance significantly attenuates its benefits [19,50]. Therefore, individualized nutritional counseling, patient education, and integration of culturally adapted dietary approaches are essential to optimize adherence and maximize therapeutic outcomes. Overall, the Mediterranean diet represents a multifaceted intervention that targets several key pathways involved in CKD progression, including inflammation, metabolic dysfunction, and gut microbiota dysregulation. Its combined renal and CV benefits support its role as a central component of comprehensive CKD management. Higher adherence to the Mediterranean diet, as assessed by validated indices such as the Mediterranean Adequacy Index, has also been associated with a reduced incidence of CV events in patients with advanced CKD [51]. However, studies specifically focused on CKD are rare, and most rely on observations or extrapolations from non-CKD population.

## 8. Dietary Modulation of Gut-Derived Uremic Toxins

Dietary interventions represent a central and modifiable strategy for regulating the production of gut-derived uremic toxins in CKD, particularly through increased intake of dietary fiber, which promotes saccharolytic fermentation and reduces the generation of protein-bound toxins such as IS and PCS [52,53]. By influencing both the composition and metabolic activity of the gut microbiota, dietary patterns directly determine the balance between saccharolytic and proteolytic fermentation pathways. This metabolic shift, as described earlier, plays a role in uremic toxin regulation and production. Consequently, dietary modulation of gut microbiota has emerged as a key therapeutic target within the gut–kidney–heart axis, with direct implications for both renal and CV outcomes.

### 8.1. Role of Dietary Fiber

Dietary fiber plays a fundamental role in shaping gut microbial metabolism and represents one of the most effective nutritional approaches for reducing the generation of uremic toxins. Increased fiber intake promotes a shift from proteolytic to saccharolytic fermentation, favoring the proliferation of beneficial bacterial taxa and enhancing overall microbial diversity [54,55]. This shift is particularly relevant in CKD, where dysbiosis is characterized by increased proteolytic activity and toxin production. The fermentation of dietary fiber by gut microbiota leads to the production of SCFAs, including butyrate, propionate, and acetate, which exert multiple protective effects. SCFAs enhance intestinal barrier integrity by strengthening tight junctions, reduce systemic inflammation through modulation of immune pathways, and improve metabolic homeostasis. In addition, SCFAs have been shown to exert direct CV benefits by improving endothelial function and reducing oxidative stress. Importantly, increased saccharolytic fermentation occurs at the expense of proteolytic pathways, resulting in reduced generation of protein-derived uremic toxins such as IS and PCS [46,56].

Results from an interventional, prospective, controlled study of 16 patients with stage 3–4 CKD—9 treated with a low-protein diet (LPD) (0.6 g/kg/day) and inulin (19 g/day), and 7 controls treated only with LPD (0.6 g/kg/day) without inulin—over a 6-month intervention showed no significant alteration of gut microbiota in either the LPD or LPD plus inulin groups. However, LPD was associated with specific shifts in bacterial taxa. In the LPD plus inulin group, there were significant reductions in serum uric acid (*p* = 0.018), CRP (*p* = 0.003), TNF-α (171.2 ± 90.3 to 116.2 ± 62.5; *p* = 0.041), and plasma nicotinamide adenine dinucleotide phosphate (NADPH) oxidase (NOX2) (0.67 ± 0.1 to 0.58 ± 0.13; *p* = 0.027) [47].

Both experimental and clinical studies have consistently demonstrated that higher dietary fiber intake is associated with lower circulating concentrations of IS and PCS in CKD patients, as well as improved inflammatory and metabolic profiles [54,55]. Dietary fiber supplementation significantly reduced serum IS (standardized mean difference [SMD] −0.34, 95% confidence interval [CI] −0.57 to −0.12; *p* < 0.01) in 200 participants in the supplementation group compared to 198 controls, based on the results of 11 RCTs analyzed in a recent meta-analysis with mild heterogeneity between studies [55]. For PCS, the supplementation group showed a significant reduction (SMD −0.22, 95% CI −0.42 to −0.02; *p* = 0.03) compared to the control group as well [55].

These findings support the central role of dietary fiber as a cornerstone of microbiota-targeted nutritional strategies aimed at reducing toxin burden and mitigating CV risk.

### 8.2. Plant-Based and Low-Protein Dietary Patterns

Plant-based and LPD patterns further enhance the reduction in gut-derived uremic toxins by limiting the availability of amino acid substrates required for proteolytic fermentation, with emerging evidence supporting plant-dominant low-protein dietary approaches as a feasible and effective strategy in CKD management [57,58]. Diets characterized by reduced intake of animal protein and increased consumption of plant-based foods decrease the generation of nitrogenous waste products and reduce the intestinal production of toxic metabolites, thereby alleviating metabolic stress in CKD [50,59]. In addition to substrate restriction, plant-based diets induce favorable shifts in gut microbiota composition [60], as previously described. This microbiota remodeling leads to a reduction in the production of uremic toxins and contributes to improved systemic metabolic and inflammatory profiles. Low-protein dietary interventions, particularly when combined with high dietary fiber intake, have been shown to reduce circulating levels of IS and PCS and to improve renal outcomes in CKD patients [46,56]. Furthermore, plant-based diets provide additional benefits through their high content of antioxidants, polyphenols, and anti-inflammatory compounds, which further contribute to vascular protection and slowing of CKD progression. Collectively, these findings underscore the importance of dietary composition in regulating gut microbiota metabolism and toxin production. Within this context, the Mediterranean diet—characterized by high intake of plant-based foods, moderate protein consumption, and abundant fiber and polyphenols—represents a practical and sustainable dietary model that integrates these beneficial components. Its ability to simultaneously promote saccharolytic fermentation, reduce toxin generation, and improve CV and renal outcomes highlights its role as a microbiota-targeted therapeutic strategy in CKD. The effects of key dietary components on gut microbiota composition, uremic toxin production, and clinical outcomes are summarized in Table 1.

## 9. Mediterranean Diet as a Microbiota-Targeted Strategy in CKD

**Figure 2:**
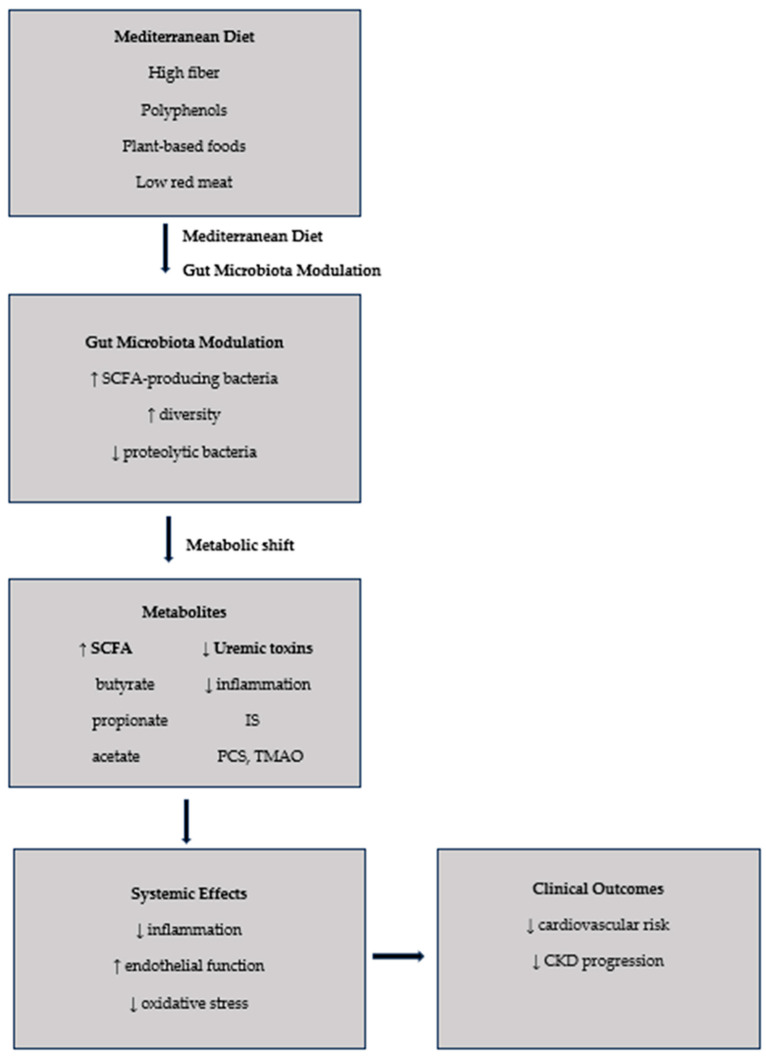
Effects of the Mediterranean diet on gut microbiota composition, uremic toxin production, and clinical outcomes in CKD. The Mediterranean diet promotes a shift in gut microbiota toward increased short-chain fatty acid (SCFA)-producing bacteria and reduced proteolytic activity. This metabolic reprogramming leads to enhanced production of beneficial metabolites (SCFAs) and decreased generation of uremic toxins, including indoxyl sulfate (IS), *p*-cresyl sulfate (PCS), and trimethylamine *N*-oxide (TMAO). These changes contribute to reduced inflammation, improved endothelial function, and decreased CV risk and CKD progression. Abbreviations: SCFA, short-chain fatty acids; IS, indoxyl sulfate; PCS, *p*-cresyl sulfate; TMAO, trimethylamine *N*-oxide; CKD, chronic kidney disease.

The Mediterranean diet has emerged as a promising microbiota-targeted strategy in CKD, integrating multiple dietary components that collectively modulate gut microbial composition, metabolic activity, and the generation of uremic toxins. By combining high intake of dietary fiber and polyphenols with reduced consumption of animal protein and processed foods, the Mediterranean diet promotes a shift in microbial metabolism from proteolytic to saccharolytic pathways, thereby reducing the production of harmful metabolites and enhancing the generation of beneficial compounds [14,15,36,39]. The mechanisms through which the Mediterranean diet modulates gut microbiota and is associated with lower levels of uremic toxins toxin production are illustrated in Figure 2.

One of the key mechanisms underlying these effects is the ability of the Mediterranean diet to reshape gut microbiota composition as previously described. The microbiota remodeling contributes to improved intestinal barrier integrity, reduced systemic inflammation, and enhanced metabolic homeostasis. Importantly, these changes in microbial composition are accompanied by significant alterations in microbial metabolic output. Diets rich in fiber and polyphenols increase the production of SCFAs, which exert anti-inflammatory and cardioprotective effects, while simultaneously reducing the generation of gut-derived uremic toxins such as IS and PCS [54,55,56]. Furthermore, reduced intake of animal-derived nutrients limits the availability of substrates for TMAO production, thereby attenuating pro-atherogenic pathways associated with CKD progression [14,15]. Although direct interventional studies specifically evaluating the effect of the Mediterranean diet on circulating levels of individual uremic toxins in the CKD population remain limited, accumulating indirect evidence strongly supports its beneficial role. Observational studies, mechanistic research, and dietary intervention trials consistently demonstrate that dietary patterns sharing key characteristics with the Mediterranean diet—such as high fiber intake, plant-based composition, and reduced protein load—are associated with lower levels of uremic toxins and improved CV and renal outcomes [38,54,55]. This body of indirect evidence highlights a critical translational concept: the Mediterranean diet may exert its protective effects not through a single pathway, but through a coordinated modulation of gut microbiota and its metabolic products. By simultaneously reducing toxin generation, enhancing beneficial metabolite production, and improving systemic metabolic and inflammatory profiles, the Mediterranean diet represents a comprehensive and biologically plausible strategy for targeting the gut–kidney–heart axis. Overall, the Mediterranean diet integrates multiple mechanisms—dietary substrate modification, microbiota remodeling, and metabolic reprogramming—that converge to reduce uremic toxin burden and CV risk in CKD. These features position it as a practical and sustainable microbiota-targeted therapeutic approach, with significant potential for clinical implementation in CKD management. Although these mechanisms have been demonstrated experimentally and clinically, direct intervention studies in participants with CKD are still lacking. Future RCTs are warranted to directly evaluate the impact of the Mediterranean diet on gut-derived uremic toxins and CV outcomes in CKD patients.

## 10. Cardiovascular Risk Reduction Through Diet–Microbiota Interactions

CV risk reduction in CKD is increasingly recognized as being closely linked to the interplay between dietary patterns, gut microbiota, and their derived metabolites. Diet–microbiota interactions influence key pathophysiological processes, including endothelial dysfunction, systemic inflammation, and vascular remodeling, which collectively contribute to the high burden of CV disease in CKD. One of the central mechanisms underlying CV protection is the improvement of endothelial function. Gut-derived uremic toxins, particularly TMAO, IS, and PCS, have been shown to impair endothelial nitric oxide availability, promote oxidative stress, and disrupt vascular homeostasis [1,2]. Dietary patterns such as the Mediterranean diet, which reduce the production of these metabolites through modulation of gut microbiota, may therefore contribute to improved endothelial function and reduced atherogenic risk. Inflammation represents another critical pathway linking gut microbiota to CV disease. Uremic toxins activate pro-inflammatory signaling pathways, including nuclear factor-κB (NF-κB), leading to increased cytokine production, endothelial activation, and immune dysregulation [1,31].

At the molecular level, pathogen-associated molecular patterns (PAMPs), such as lipopolysaccharide (LPS) and lipoteichoic acid (LTA), activate toll-like receptors (TLRs), triggering downstream signaling pathways and promoting the release of pro-inflammatory cytokines, including interleukin-1β (IL-1β), tumor necrosis factor-α (TNF-α), and interleukin-6 (IL-6). In parallel, SCFAs interact with TLRs through G protein–coupled receptors (GPR41, GPR43, and GPR109A), mediating transmembrane signaling via heterotrimeric G proteins. This interplay regulates key inflammatory and immune pathways, including nuclear factor kappa B (NF-κB), mitogen-activated protein kinase (MAPK), peroxisome proliferator-activated receptor (PPAR), and NOD-like receptor family pyrin domain-containing 3 (NLRP3) inflammasome activation, and therefore contributes to the balance between pro- and anti-inflammatory responses [61].

In contrast, dietary interventions rich in fiber and polyphenols promote the production of SCFAs, which exert anti-inflammatory effects and help restore immune balance. This shift toward an anti-inflammatory profile is associated with reduced systemic inflammation and improved CV outcomes [3,4]. Vascular stiffness and calcification are also significantly influenced by gut-derived metabolites. Elevated levels of TMAO and protein-bound uremic toxins have been associated with increased arterial stiffness, vascular calcification, and impaired vascular compliance [3,31]. These structural changes contribute to increased pulse wave velocity and heightened CV risk in CKD patients. Dietary modulation of gut microbiota, particularly through Mediterranean and plant-based dietary patterns, may attenuate these processes by reducing toxin generation and improving vascular function. Importantly, the integration of dietary strategies with microbiota-targeted mechanisms provides a comprehensive framework for CV risk reduction in CKD. By simultaneously improving endothelial function, reducing inflammation, and mitigating vascular stiffness, diet–microbiota interactions represent a key therapeutic axis.

Mentioned interconnected mechanisms represent the gut–kidney–heart axis main components in linking dietary patterns and microbiota-derived metabolites to CKD population CV outcomes. 

This integrative approach highlights the potential of the Mediterranean diet as a non-pharmacological strategy for addressing the complex pathophysiology of CV disease in CKD.

It is important to note that robust evidence from CKD-specific RCTs demonstrating a causal relationship is limited, and most available evidence comes from mechanistic and observational studies.

## 11. Emerging Therapeutic Perspectives Targeting the Gut–Kidney Axis

The growing recognition of the gut–kidney axis as a central pathway in the pathophysiology of CKD has led to increasing interest in novel therapeutic strategies aimed at modulating gut microbiota composition and metabolic activity. These approaches focus on reducing the production of uremic toxins, restoring microbial balance, and improving both renal and CV outcomes [8]. Among these strategies, prebiotics have emerged as a promising intervention due to their ability to selectively stimulate the growth of beneficial gut bacteria. Prebiotic compounds, particularly non-digestible dietary fibers, enhance saccharolytic fermentation and promote the production of SCFAs, which improve intestinal barrier integrity, reduce systemic inflammation, and suppress the generation of protein-derived uremic toxins such as IS and PCS [7,8]. These effects are particularly relevant in CKD, where dysbiosis is characterized by increased proteolytic activity and toxin production. Probiotics represent another therapeutic approach aimed at directly modifying gut microbiota composition through the administration of beneficial microbial strains. Experimental and clinical studies suggest that probiotic supplementation may reduce circulating levels of uremic toxins, modulate immune responses, and improve metabolic profiles in CKD patients [6,7]. However, the clinical efficacy of probiotics remains variable, likely due to differences in strains, dosages, and patient populations, highlighting the need for standardized protocols and well-designed RCTs. In addition to these interventions, personalized nutrition has gained attention as a future direction in the management of CKD. Advances in microbiome research and omics technologies have enabled a more precise understanding of individual variability in gut microbiota composition and metabolic responses to dietary interventions. Personalized dietary approaches, tailored to an individual’s microbiome profile, metabolic status, and clinical characteristics, may optimize the modulation of uremic toxin production and enhance therapeutic outcomes [6,8]. Importantly, these emerging strategies should not be viewed as isolated interventions but rather as complementary components of a broader, integrative approach targeting the gut–kidney–heart axis. In this context, the Mediterranean diet provides a practical foundation upon which additional microbiota-targeted therapies, such as prebiotics and probiotics, may be integrated. This combined strategy holds significant potential for reducing uremic toxin burden, improving CV risk, and slowing CKD progression. Overall, emerging therapeutic approaches targeting the gut–kidney axis highlight a paradigm shift from conventional treatment strategies toward microbiota-centered interventions. Future research should focus on validating these approaches in large-scale clinical trials and integrating them into personalized treatment frameworks for patients with CKD.

## 12. Limitations of Current Evidence

Despite the growing body of evidence supporting the role of diet–microbiota interactions in modulating uremic toxin production and CV risk in CKD, several important limitations should be acknowledged. These limitations primarily relate to the scarcity of direct interventional studies, heterogeneity in study designs, and variability in dietary adherence and microbiota responses. One of the major limitations is the limited availability of studies directly evaluating the effect of the Mediterranean diet on circulating levels of specific gut-derived uremic toxins. While numerous studies have demonstrated associations between dietary patterns, microbiota composition, and toxin generation, most of the current evidence remains indirect or derived from observational analyses [8,15]. As a result, the causal relationship between Mediterranean diet adherence and reduction in specific uremic toxins, such as IS, PCS, and TMAO, is not yet fully established. In addition, significant heterogeneity exists across available studies in terms of patient populations, CKD stages, dietary assessment methods, and microbiota analysis techniques. Differences in study design, duration of dietary interventions, and endpoints further complicate the interpretation and comparison of results [2,8]. This heterogeneity limits the ability to draw definitive conclusions and highlights the need for standardized methodologies in future research. Another important limitation relates to variability in dietary adherence and real-world implementation of Mediterranean dietary patterns. Adherence to dietary interventions can vary substantially among patients due to cultural, socioeconomic, and clinical factors, which may influence both microbiota composition and clinical outcomes [19,50]. This variability represents a significant challenge for translating findings from controlled settings into routine clinical practice. Furthermore, much of the mechanistic evidence linking gut-derived metabolites to CV outcomes is based on experimental or associative data rather than large-scale RCTs. For example, while metabolites such as TMAO have been consistently associated with increased CV risk, direct evidence demonstrating that dietary modulation leads to clinically meaningful reductions in CV events remains limited [2]. Taken together, these limitations underscore the need for well-designed, large-scale RCTs that specifically investigate the impact of Mediterranean diet interventions on gut-derived uremic toxins and CV outcomes in CKD patients. Addressing these gaps will be essential for translating current mechanistic insights into evidence-based clinical recommendations.

There are several limitations of this narrative review that need to be addressed. Primarily, due to its narrative nature, it is susceptible to selection bias, although the search strategy and inclusion and exclusion criteria are defined. A substantial proportion of the available evidence comes from observational studies, preclinical models, and indirect dietary interventions, which limits the ability to establish causal relationships among Mediterranean diet adherence, gut microbiota modulation, and reductions in specific uremic toxins. There is considerable heterogeneity across studies in patient populations, stages of CKD, dietary assessment methods, and microbiota analysis techniques, complicating direct comparisons and limiting the generalizability of findings. Dietary adherence is a critical and variable factor that is often insufficiently controlled or reported, potentially affecting the consistency of observed outcomes. Furthermore, while mechanistic pathways linking gut-derived metabolites to cardiovascular risk are well supported experimentally, there is still a lack of large-scale randomized controlled trials directly demonstrating that dietary modulation leads to clinically meaningful reductions in cardiovascular events in CKD populations.

## 13. Conclusions

CKD is characterized by a complex interplay between intestinal dysbiosis, accumulation of gut-derived uremic toxins, and increased CV risk, collectively defined as the gut–kidney–heart axis. This review highlights the central role of gut microbiota as a key mediator linking dietary patterns to both renal and CV outcomes. In particular, the shift from saccharolytic to proteolytic fermentation in CKD promotes the generation of toxic metabolites such as IS, PCS, and TMAO, which contribute to endothelial dysfunction, inflammation, and vascular remodeling. The Mediterranean diet emerges as a promising microbiota-targeted strategy that may modulate these pathogenic pathways. With its high content of dietary fiber, polyphenols, and plant-based components, this dietary pattern may promote beneficial microbial shifts, enhance short-chain fatty acid production, and reduce the generation of uremic toxins. From a clinical perspective, incorporating Mediterranean dietary principles into CKD management represents a feasible and non-pharmacological strategy with the potential to simultaneously address multiple pathogenic pathways.

However, most available evidence comes from observational and mechanistic studies, while direct interventional data in CKD populations remain limited.

Future research should focus on well-designed RCTs and personalized nutrition approaches to further elucidate the role of diet–microbiota interactions and to translate these findings into evidence-based clinical practice.

## Figures and Tables

**Table 1:** Effects of Dietary Components on Gut Microbiota Composition, Uremic Toxin Production, and CV Outcomes in CKD.

Component	Microbiota Effect	Metabolite Effect	Clinical Impact
Fiber	↑ microbial diversity; ↑ saccharolytic bacteria	↑ SCFAs (butyrate, propionate); ↓ IS, PCS	↓ systemic inflammation
Polyphenols	selective stimulation of beneficial taxa (prebiotic-like effect)	↑ SCFAs; ↓ oxidative metabolites	↓ oxidative stress
Reduced red meat intake	↓ proteolytic bacteria; ↓ TMA-producing taxa	↓ TMAO	↓ atherosclerotic risk
Plant-based diet	↑ beneficial taxa; ↓ proteolytic fermentation	↓ IS, PCS, TMAO	↓ CKD progression and CV risk
Mediterranean diet	integrated modulation of microbiota composition and function	↓ IS, PCS, TMAO; ↑ SCFAs	↓ CV risk; improved cardiometabolic profile

Abbreviations: SCFAs, short-chain fatty acids; IS, indoxyl sulfate; PCS, *p*-cresyl sulfate; TMAO, trimethylamine *N*-oxide; CKD, chronic kidney disease; CV, cardiovascular.

## Data Availability

No new data were created or analyzed in this study. Data sharing is not applicable.

## Abbreviations

The following abbreviations are used in this manuscript:

CKD	chronic kidney disease
CV	cardiovascular
IS	indoxyl sulfate
PCS	*p*-cresyl sulfate
TMAO	trimethylamine *N*-oxide
SCFA	short-chain fatty acids
eGFR	estimated glomerular filtration rate
RCTs	randomized controlled trials
